# Validation of subscales of the Severe Asthma Questionnaire (SAQ) using exploratory factor analysis (EFA)

**DOI:** 10.1186/s12955-020-01593-9

**Published:** 2020-10-09

**Authors:** Joseph W. Lanario, Michael E. Hyland, Andrew Menzies-Gow, Adel H. Mansur, James W. Dodd, Stephen J. Fowler, Rupert C. Jones, Matthew Masoli

**Affiliations:** 1 University of Plymouth, Plymouth, UK; 2 Plymouth Marjon University, Plymouth, UK; 3 Royal Brompton Hospital, London, UK; 4 Heartlands Hospital, University Hospitals Birmingham, University of Birmingham, Birmingham, UK; 5 Academic Respiratory Unit, Southmead Hospital, North Bristol Hospital Trust, Bristol, UK; 6 School of Biological Sciences, Faculty of Biology, Medicine and Health, University of Manchester, Manchester Academic Health Science Centre and NIHR Manchester Biomedical Research Centre, Manchester University Hospitals NHS Foundation Trust, Manchester, UK; 7 Royal Devon and Exeter Hospital, University of Exeter, Exeter, UK

**Keywords:** Asthma, Quality of life, Measurement, Exploratory factor analysis

## Abstract

**Background:**

The Severe Asthma Questionnaire (SAQ) is a health related quality of life (HRQoL) questionnaire validated for use in severe asthma. It is scored using the mean value of 16 items (SAQ score) in addition to a single item global rating of HRQoL (SAQ-global). The aim was to validate clinically relevant subscales using exploratory factor analysis (EFA).

**Methods:**

The SAQ was completed, along with measures of asthma control and EQ5D-5L by patients attending six UK severe asthma centres. Clinical data were included in the analysis. EFA using principal axis factoring and oblimin rotation was used to achieve simple structure of data.

**Results:**

460 patients with severe asthma participated, 65% women, mean age 51 (16–83) years. A three factor solution achieved best fit and showed that the SAQ items formed three distinct but inter-correlated groups of items where items were grouped in a way that was consistent with item content. The three subscales were differentially associated with clinically relevant variables (lung function and mood). Males and females interpreted the question of night disturbance in different ways.

**Conclusions:**

This paper provides a template for best practice in the use of EFA when validating HRQoL subscales. The SAQ can be scored as three subscales with content reflecting three different constructs people with severe asthma use when making judgements about their lives. The subscale ‘My Life’ assesses the impact of severe asthma on different life activities, ‘My Mind’ assesses the perceived emotional impact and ‘My Body’ the impact of extra-pulmonary symptoms and side effects.

## Background

Validated health-related quality of life (HRQoL) questionnaires are used in clinical practice and research to evaluate the impact of disease and/or treatment responses. They consist typically of several items the responses to which are aggregated to form an overall HRQoL score. Subscales can be formed from groups of items as subscales provide information that can be useful in clinical trials and clinical practice.

Guidelines for validating questionnaires recommend a two stage process where content validity is followed by construct validity [1], but these recommendations were published after the publication of four asthma specific HRQoL questionnaires. Validation of the asthma quality of life questionnaire’s (AQLQ) subscales is based only on content validity [2, 3] as the subscales are formed by grouping items on the basis of an examination of content alone. However, in three other asthma specific HRQoL questionnaires subscales are validated by both content validity and construct validity. This is done by showing that items initially grouped on the basis of content have similar statistical properties, using either principal component analysis [4, 5] or principal factor analysis [6]. The advantage of construct validation is that it can show whether patients’ interpretation of the meaning of items is the same as that of the researchers.

The number of subscales in existing asthma specific HRQoL varies between three [5], four [2], five [6] and six [4], but despite this variation, there is consensus that activity restriction and mood should be measured in different subscales. Activity-related items are assessed in subscales labelled ‘activity limitation’ [2], ‘activity’ [5], and ‘activities’ and ‘avoidance’ [6]. Items relating to the emotional impact of asthma are assessed in subscales labelled ‘emotional function’ [2], ‘mood’ [4] and ‘distress’ and ‘preoccupation’ [6].

The Severe Asthma Questionnaire (SAQ) is the only validated HRQoL questionnaire for specifically severe asthma [7]. It comprises 16 inter-correlated items measuring the impact of disease and medical interventions, and the mean of those items forms the SAQ score [8]. In addition, the questionnaire has a single question measuring the impact of disease and its treatment on the patient’s overall perception of quality of life, the SAQ-global score. Content validity for the questionnaire was established in two qualitative studies [7, 9], and construct validity for the SAQ score demonstrated by factor analysis. The content of the16 items fall into three categories. Items 1–7 ask patients to rate the impact of their asthma and its treatment on seven different types of life activity, and have content consistent with items in the activity subscales of earlier questionnaires. Items 8–11 ask patients about various aspect of mood and have content consistent with that in the emotional subscales of earlier questionnaires. Items 12–16 assess the impact on life of extra-pulmonary symptoms and side effects. These last five items measure quality of life deficits that are typically found only in severe asthma where qualitative research shows them to play a major role [7, 9–11]. They are rare in mild and moderate asthma and have limited representation in earlier asthma specific HRQoL questionnaires [9]. Content derived subscales for the SAQ and one consistent with earlier scales would therefore be based on three subscales (a) impact on life’s activities (b) impact on emotional well-being, and (c) impact of extra-pulmonary symptoms including those caused by side effects of treatment.

Although there is a rationale for having three subscales of the SAQ on the basis of content, that subscale structure has not been construct validated. Subscales of HRQoL scales are useful because they provide more detail about the patient’s experience, and this greater detail facilitates communication with the patient as well providing more nuanced information about HRQoL change in clinical trials. The aim of this study is to provide construct validation of the subscales of the 16 items of the SAQ by showing that the different subscales are associated with different constructs. In this study, construct validity is achieved by providing evidence that (a) the 16 items fall into statistically distinct clusters; (b) that items in the statistically formed subscales are consistent with classification based on content; (c) that the subscales formed from these clusters have different associations with clinically relevant variables and therefore provide additional information compared to the overall scores.

## Methods

### Design

This was a cross-sectional study with questionnaire and clinic data each collected at one time point.

### Participants

Patients aged ≥ 16 years of age and diagnosed with severe asthma as defined by the ERS/ATS guidelines were invited to participate [12]. Participants were recruited from six UK severe asthma centres and were excluded if they were diagnosed with another condition that significantly contributed to their respiratory health, e.g. lung cancer, heart disease or chronic obstructive pulmonary disease.

### Patient reported outcome measures

#### Severe Asthma Questionnaire (SAQ)

The SAQ consists of 16-items scored from 1 to 7, with a higher score indicating better quality of life. The mean of the 16 items is calculated to provide the SAQ score. The SAQ also contains a separate, Borg-type scale ranging from 0–100 and based on the Global Quality of Life Questionnaire [13] which provides the SAQ-global score [8].

#### Asthma Control Test (ACT)

The ACT consists of five asthma symptom and medication use items, which are totalled to provide an indication of asthma control. The sum of the five items, scale-5, is calculated to give the ACT score, with a higher number indicating better asthma control [14].

#### Asthma Control Questionnaire-6 (ACQ-6)

The ACQ consists of six items, five concerning asthma symptoms and one on daily use of rescue bronchodilator. Patients respond to these items on a 0–6 scale (0 = no impairment, 6 = maximum impairment). The mean of the six items is calculated to provide the ACQ-6 score with a lower number indicating better asthma control [15].

#### EQ-5D-5L and mood measurement

The EQ-5D-5L consists of 5 items scored from 1 to 5 with a higher score indicating greater impairment, and a 0–100 visual analogue score, the EQ-5D VAS [16]. Index scores are calculated using the 2012 value set for England [17] and these index scores are presented here. For this study we used item 5 of the questionnaire as a proxy measure of mood. Participants indicate the degree to which they feel “anxious or depressed” on a five point scale of severity.

### Clinical data

Clinical data included body mass index (BMI) and asthma severity as measured by the following items: GINA treatment step, spirometry (forced expiratory volume in 1 s (FEV_1_) and FEV_1_% predicted), prednisolone dose (mg/day), health care utilisation in the last 12 months including number of hospital admissions, emergency department visits and exacerbations requiring oral corticosteroids (OCS). An estimate of cumulative OCS exposure (mg/year) was calculated by multiplying a patient’s maintenance OCS dose by 365 and adding an estimated use of OCS following each exacerbation. British Thoracic Society and GINA guidelines suggest that 40 mg of prednisolone for 7 days should be prescribed for the treatment of exacerbations [18]. This equates to 280 mg of OCS per exacerbation.

### Procedure

Patients with severe asthma at five specialist treatment centres were approached for recruitment to this study. Questionnaires were completed in clinic once written informed consented was given. Spirometry was conducted either at the time of questionnaire completion or the most recent within the previous 6 months. Participating sites collected either ACT or ACQ data as a measure of asthma control for this study as per their normal clinical practice. The same data collected for a previous study [8] from a sixth specialist centre were also included for analysis.

### Ethical approval

This study received ethical approvals from the Research Ethics Committee/Health Research Authority (REC reference: 19/WA/0011, IRAS project ID: 250167) and was sponsored by University Hospitals Plymouth NHS Trust. Data from a previous study received ethical approval number 16/NE/0188, IRAS ID: 207601) [8].

### Statistical analysis

Exploratory factor analysis (EFA) is a statistical procedure that can be used to make inferences about underling causal structures. The procedure is based on the assumption that correlations between variables is due to a common cause, referred to mathematically as a factor (i.e., causal factor) and psychologically as a construct (i.e., psychological construct.) In the case of patient reported outcomes, the constructs are dimensions of meaning that are responsible for the way patients interpret and respond to the individual items of a questionnaire. People can use many different dimensions of meaning to evaluate their outcomes, so the aim of the technique is to identify the main dimensions that drive response to individual items. Factor solutions that achieve a ‘simple structure’ [19, 20] indicate that those main dimensions have been identified and therefore provides a good description of the underlying dimensions of meaning used to interpret the items of a questionnaire. However, people interpret any item of a questionnaire by using one or more dimensions of meaning, and so discovery of the main dimensions of meaning is aided if the items tend to be specific to different meaning dimensions. Factor analysis of patient reported outcomes is therefore a way of exploring the meaning of a questionnaire but that exploration depends on the items of the questionnaire. The meaning of simple structure and the rationale for choosing the factor parameters for this analysis are described below.

There are two main forms of data extract: principal component analysis and factor analysis. Principal component analysis is a simpler and older form of analysis that became popular when computers were slower and is the default option in many statistical packages. Principal component analysis is a method of data reduction only, it does not distinguish between unique and shared variance and therefore does not identify causal factors (psychological constructs). The method risks overestimating variance. Factor analysis analyses only shared variance and in so doing provides information about underlying causal structures, it does not inflate estimates of variance and for most purposes is the recommended form of extraction [20]. We used factor analysis rather than principal component extraction because we wanted to identify causal constructs and estimate variance, and we used principal axis factor analysis as a commonly used type of factor analysis [20].

EFA is an exploratory tool that provides choice in the numbers of factors to be extracted. When used for subscale construction in HRQoL, the primary determinant of factor number and hence subscale number is a number that is both theoretically plausible and clinically useful. If that number produces a simple structure (see later), then that number can be accepted as the final solution. If that number fails to produce a simple structure, then alternatives should be considered. In our case, a plausible and useful number based on content is that there should be three factors, corresponding to activity, emotion and extra-pulmonary symptoms.

There are several driven methods of determining factor number that can be used in addition to the primary, theoretical determination, but these methods typically produce different results and are therefore advisory only [19]. The eigenvalue is a measure of variance explained, and because of the way factors are extracted eigenvalues decrease with the number of factors extracted. The default setting in many statistical packages is to select the number of factors with eigenvalues greater than one (the Kaiser–Guttman rule) [21]. Because eigenvalues increase with the number of items analysed this method provides limited information and is widely held to be the least useful data driven method of advising on factor number [19, 20]. However, the overall pattern of all eigenvalues is useful not only by providing data for another, widely recommended test of factor number, the scree test. The scree test requires inspection of the eigenvalues to determine the point at which eigenvalues reduce in a similar way–the analogy is with the scree at the bottom of a cliff.

Once the number of factors is set, principal axis factoring coupled with rotation provides a solution capable of interpretation. The technique of rotation can be done either by forcing the factors to be uncorrelated (called orthogonal rotation, e.g., varimax) or allowing the factors to be correlated (called oblique rotation, e.g., oblimin, promax), each type of orthogonal or oblique rotation having slightly different properties. Orthogonal rotation should be used only when uncorrelated factors are predicted on theoretical grounds or when there is evidence from an earlier oblique rotation that the factors are largely uncorrelated. Varimax (i.e., orthogonal) rotation became popular through its use in psychology where there was a theoretical requirement for personality factors to be uncorrelated [22], but this form of rotation is often used incorrectly in situations where factors may be correlated. In the present case, factors are predicted to be correlated as the three content derived domains of the SAQ all form part of the overall HRQoL. Promax and oblimin are commonly used forms of oblique rotation, promax being computationally simpler than oblimin, oblimin being the preferred form [20] and that which was used here.

EFA produces a factor matrix where each item of a questionnaire has a value, called a loading, on each of the factors. The item loadings vary between − 1 and 1 and can be considered equivalent to correlations between the item and the artificial variable represented by the factor. We adopted the convention that items that load at or greater than 0.3 should be allocated to that factor [19, 20]. Orthogonal rotations produce only one factor matrix whereas oblique (i.e., correlated) rotations produce two matrices, the structure matrix and the pattern matrix. The pattern matrix expresses the relationship between items and a factor after removing the effect of the correlations between the factors, and therefore provides a clearer picture of the separation of items between factors, should that be the case, compared to the alternative, the structure matrix. However, by removing the correlations between factors, only the factor loadings of the structure matrix but not of the pattern matrix can be considered equivalent to a correlation with an artificial variable. Therefore, in order to interpret the pattern matrix it is necessary to know the degree of correlation between the factors produced by the rotation. These factor correlations are reported separately from the pattern matrix, and are similar but not identical to subscale correlations because factor correlations are based on response to weighted items whereas subscales are based on unweighted items [19].

Rules for sample size for EFA have largely disappeared because sample size depends to some extent on the data though a common rule of thumb is a ratio of 10:1 participants to items [19]. Adequacy of sample size can be checked statistically. The solution provided by any EFA depends on the correlation matrix between the variables. Differences in that matrix resulting from low correlations and small sample sizes can produce large differences in solution, i.e., factor instability. The Kaiser–Meyer–Olkin measure of sampling adequacy provides a way of measuring the level of factor stability. The Kaiser–Meyer–Olkin varies between zero and one, values above 0.8 indicating that the factor solution is likely to be stable, and above 0.9 highly stable. However, if sample size allows, factor stability can be checked by separate analysis of subgroups. In the analysis conducted here, we examined factor solutions for males and females separately, a technique that also checks that males and females interpret every item in the same way.

The aim of an EFA, as a statistical tool, it to find a solution where there is a simple structure to the data. Simple structure is summarised as “item loadings above 0.30, no or few item crossloadings, no factors with fewer than three items” [20]. Validation of HRQoL subscales has an additonal requirement, that the subscales so produced are both theoretically plausiable in terms of content as well as clinically useful. An EFA solution producing 10 subscales may achieve simple structure but is unlikely to have much clinical use. Cross-loading items (i.e., where the loading is > 0.3 on more than one factor) indicate either that response to the item is affected by more than one construct, or that the solution provides a poor fit for data. Either way, the presence of cross-loading items is undesirable and absence of all but a bare minimum of cross-loading items is a primary requirement for construct validation of subscales of a HRQoL questionnaire [20]. Validation requires a “clean” factor matrix, namely one where there is good separation between loadings for every item. An item that loads 0.32 on one factor and 0.25 on another is a poor item. Items with loadings of 0.32 and 0.01 and 0.25 and 0.60 are acceptable, but the goal is for the largest possible separation.

Although a HRQoL questionnaire may fail to provide validated subscales according to the criteria described above, the overall scale score can still be used. It is almost inevitable that all the items of HRQoL questionnaires will load on the first unrotated factor. This is because, in general, HRQoL deficits in a population increase with severity and so the first factor unrotated factor is simply a severity factor. An HRQoL item must by definition be related to health and it would be unusual if an item failed to correlate with overall severity. Subscale construct validation by EFA is more demanding as it requires specificity of items to constructs, rather than specificity to severity.

Following EFA, subscales were constructed on the basis of the factor loadings by taking the mean of items loading on any factor. The relationship between the subscales and other variables was examined using Pearson correlations. EFA and correlations were conducted using SPSS version 25. Tests of difference between correlations were carried out using Psychometrica (https://www.psychometrica.de/correlation.html).

## Results

The total sample size was 460 consisting of data from 160 participants who provided data for a previous validation study [8] and 300 participants who provided new data. Two hundred and ninety-nine (65%) of the participants were female. Further patient demographics are shown in Table 1 and the mean questionnaire scores are shown in Table 2.

**Table 1:** Participant demographics

	N	Mean (CI)	n (%)
Age, yrs	460	51 (50–53)	
Female, n	460		299 (65)
FEV_1_, L	457	2.12 (2.05–2.20)	
FEV_1_, % predicted	454	71.75 (69.79–73.71)	
Caucasian, n	460		416 (91)
BMI, kg/m^2^	459	31.10 (30.39–31.81)	
Prescribed maintenance OCS, n	460		218 (47)
Exacerbations in the last 12 months requiring OCS, n	460	3.74 (3.35–4.12)	
Emergency Department visits	460	0.91 (0.66–1.15)	
Hospital visits	460	0.65 (0.43–0.87)	
Cumulative prednisolone, mg/yr	460	3148 (2814–3483)	
Receiving biologics, n	456		180 (39)

Sample sizes vary as a function of data availability

**Table 2:** Mean questionnaire scores (95% confidence intervals)

	n	Mean
SAQ score	449	3.99 (3.84–4.14)
SAQ My Life	449	4.16 (3.99–4.32)
SAQ My Mind	449	4.04 (3.87–4.21)
SAQ My Body	449	3.58 (3.43–3.73)
SAQ-global score	452	53.88 (51.66–56.10)
ACQ score	258	2.68 (2.50–2.86)
ACT total	200	14.32 (13.49–15.14)
EQ-5D-5L	381	0.69 (0.67–0.72)
EQ-5D VAS	383	61.03 (58.74–63.13)
EQ-5D-5L item 5-anxiety/depression	381	2.12 (2.06–2.89)

Sample sizes vary as a function of data availability

The Kaiser–Meyer–Olkin measure of sampling adequacy was 0.96 for the total sample and was 0.94 for both the male and female subgroup analyses. For the total sample, the first five eigenvalues were 10.5, 1.1, 0.87, 0.64 and 0.45. All 16 items loaded > 0.64 on the first unrotated factor of a principal axis factor analysis. The pattern matrix is shown in Table 3. The factor correlations were: factors 1 and 2 *r* = 0.70, factors 1 and 3 *r* = 0.73, factors 2 and 3 *r* = 0.67.

**Table 3:** Factor loadings of the pattern matrix of a principal axis factor extraction

	Factor 1	Factor 2	Factor 3
	My Life	My Mind	My Body
1. My social life	0.88	− 0.05	0.10
2. My personal life	0.85	0.10	− 0.10
3.My leisure activities	0.83	− 0.07	0.13
4. My jobs around the house	0.97	− 0.10	0.02
5. My work or education	0.76	0.05	0.07
6. My family life—how it affects me	0.84	0.15	− 0.07
7. My family life—how it affects others	0.66	0.28	− 0.03
8. Depression	0.08	0.86	0.03
9. Irritable	0.11	0.73	0.10
10. Anxiety in general	− 0.06	0.94	0.05
11. Worry that asthma may get worse	0.15	0.42	0.27
12. Worry about long term side effects of medicines	0.04	0.18	0.52
13. Getting tired	0.23	0.115	0.56
14. Problems at night	0.47	− 0.01	0.41
15. The way I look	− 0.05	− 0.00	0.89
16. Problems with food	0.10	0.10	0.64

Fifteen of the 16 items loaded on only one of the three factors with item grouping consistent with the content derived domains. Item 14 (night disturbance) loaded on two factors: factor 1 and factor 3. When EFA was repeated separately for males and females, then any item loading on a factor in the overall analysis was replicated in these sub-analyses, with one exception. For males item 14 loaded only on factor 1 (0.67) but not on factor 3 (0.29) or 2 (− 0.10), whereas for females item 14 loaded on factor 1 (0.41) and on factor 3 (0.41) but not on factor 2 (0.57). These results indicate that for the cross-loading item 14, males and females respond in different ways.

Table 4 shows the correlations between clinically relevant variables and the three subscales created from the mean of items allocated to that subscale. Table 5 shows the correlations between all questionnaires. Together these two tables illustrate differences in correlations between subscales and theoretically relevant variables. Using tests of difference between correlations, the correlation between FEV_1_% predicted and My Life was significantly different (*p* = 0.016) from the correlations between FEV_1_% predicted and either My Mind or My Body. The correlation between Anxiety/depression and My Mind was significantly different (*p* < 0.001) from the correlations between Anxiety/depression and either My Life or My Body.

**Table 4:** Correlations between different scores of the SAQ, EQ-5D-5L Index value and other variables

	FEV_1_% predicted	BMI	Cumulative prednisolone, mg/yr	Exacerbations in the last 12 months requiring OCS	Hospital admissions in the last 12 months
SAQ score	0.23** (443)	− 0.28** (448)	− 0.34** (449)	− 0.37** (449)	− 0.17** (449)
SAQ My Life	0.29** (443)	− 0.29** (448)	− 0.35** (449)	− 0.37** (449)	− 0.16** (449)
SAQ My Mind	0.15** (443)	− 0.21** (448)	− 0.23** (449)	− 0.33** (449)	− 0.16** (449)
SAQ My Body	0.15** (443)	− 0.28** (448)	− 0.34** (449)	− 0.33** (449)	− 0.13** (449)
SAQ global score	0.28** (446)	− 0.25** (451)	− 0.37** (452)	− 0.36** (452)	− 0.23** (452)
EQ-5D-5L Index value	0.22** (375)	− 0.36** (380)	− 0.31** (381)	− 0.25** (381)	− 0.19** (381)
EQ-5D VAS	0.24** (377)	− 0.24** (382)	− 0.34** (383)	− 0.36** (383)	− 0.18** (383)

^*^p < 0.05; **p < 0.01

**Table 5:** Correlations (N^a^) between study questionnaires

	SAQ score	SAQ My Life	SAQ My Mind	SAQ My Body	SAQ global score	EQ-5D-5L Index value	EQ-5D-5L item 5-anxiety/depression	EQ-5D VAS
SAQ My Life	0.95** (449)							
SAQ My Mind	0.90** (449)	0.77** (449)						
SAQ My Body	0.91** (449)	0.80** (449)	0.77** (449)					
SAQ global score	0.77** (441)	0.79** (441)	0.64** (441)	0.66** (441)				
EQ-5D-5L Index value	0.72** (374)	0.73** (374)	0.64** (374)	0.59** (374)	0.66** (376)			
EQ-5D-5L item 5–Anxiety/Depression	− 0.64** (376)	− 0.54** (376)	− 0.73** (376)	− 0.56** (376)	− 0.50** (378)	− 0.72** (381)		
EQ-5D VAS	0.73** (375)	0.74** (375)	0.63** (375)	0.62** (375)	0.79** (379)	0.72** (379)	− 0.52** (381)	
ACQ score	− 0.75** (253)	− 0.79** (253)	− 0.62** (253)	− 0.60** (253)	− 0.77** (256)	− 0.66** (240)	0.48** (241)	− 0.73** (240)
ACT total	0.71** (195)	0.72** (195)	0.62** (195)	0.64** (195)	0.68** (194)	0.59** (139)	− 0.50** (140)	0.63** (141)

^**^p < 0.01

^a^N varies due to availability of data

## Discussion

**Fig. 1:**
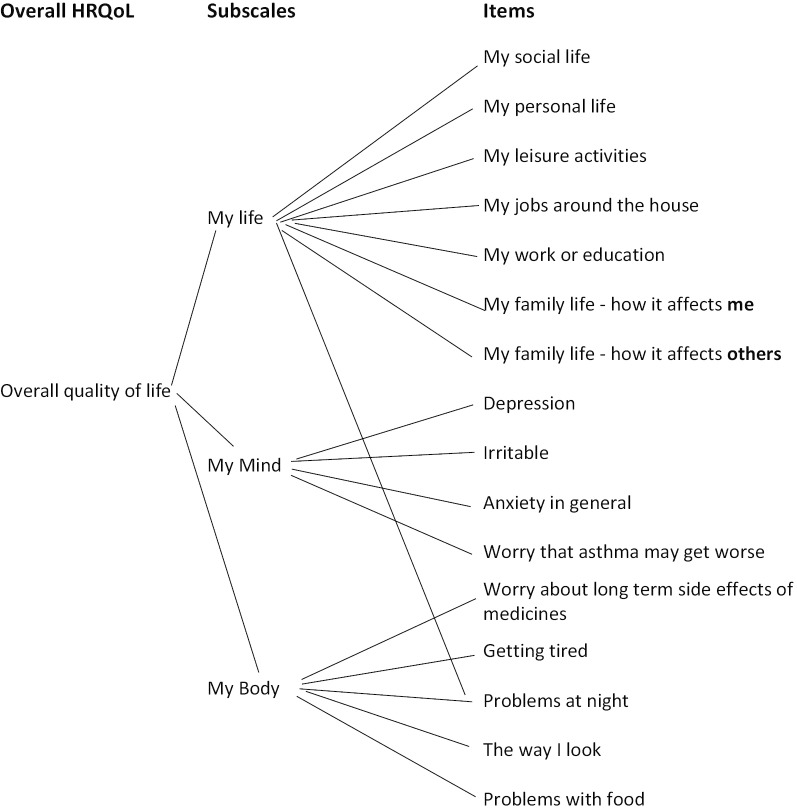
Relationship between items of the SAQ and three subscales

According to current recommendations, measures of patient reported outcomes should be validated first by content validity and then by construct validity [1]. In the case of subscales it is possible to group items on the basis of content alone, and this is a feature of some of the earlier scales [2]. Construct validation provides an additional level of certainty by showing that there is a statistical basis for grouping items. In this study we used EFA to show that the 16 items of the SAQ fall into three groups, with all but one the items loading only on one factor, that one item (night disturbance) loading on two factors and therefore not performing according to prediction. On the basis of item content, of the single loading items, the groups of items are given the domain labels, *My Life* as the items refer to activities and other aspects of a person’s life, *My Mind* as the items refer to self-perceptions of mental state and *My Body* as the items refer to the perceived impact of extra-pulmonary symptoms including side effects on the body. The relationship between items and subscales is shown in Fig. 1.

The night disturbance item cross-loads on the My Life and My Body factors, but males and females interpret the question differently. For females, the night disturbance item loads equally on the My Life and My Body factors, showing that for females night disturbance limits daily activity as well as adversely affecting bodily perceptions (e.g., fatigue and appearance). For males, night disturbance loads on the My Life factor and just misses significance on the My Body factor, indicating that for males the meaning of night disturbance is primarily, but not exclusively, in terms of limitation to daily activities. The night disturbance item (item 14) is scored to contribute to both the My Life and My Body subscales, consistent with the data from the total sample.

The factor structure obtained in this analysis can be compared to that obtained with the earlier three HRQOL scales for mild and moderate asthma that also used component or factor analysis. Although all three scales provide evidence of a distinction between activity restriction and emotional impact, the results are very different. Only one study that used oblique rotation achieved best fit producing five factors with minimal cross-loading items [6]. One using varimax rotation and produced a very poor fit of six factors with 28 out of 31 items cross-loading [4]. One reported “good separation” of three factors but without reporting any factor loadings [5] or any other data. One used the scree test to determine the number of factors but without presenting the eigenvalues on which the test is based [4], one reported eigenvalues and, after demonstrating that the scree test could not be used, used pragmatic examination to give five factors [6], and one used three factors on the basis of content alone [5]. By definition all HRQoL items should correlate with health, and because of this items form a hierarchical structure where all items load on a first factor. In the present data, all items loaded > 0.6 on a first factor. The result is that in all HRQoL questionnaires the scree test is likely to indicate a one factor solution, and will do so long as the items are good measures of health. High loadings on a first principal axis factor are also a feature of some biomarkers because they also reflect an underlying dimension of health [23]. Although the scree test is recommended instead of the Kaiser–Guttman test [16] and is a useful statistical guide for factor number when factors are uncorrelated or weakly correlated, it cannot be used for determining the number of factors in the case of HRQoL questionnaires because of the hierarchical structure of the items. Nevertheless, whether or not a scree test is used, eigenvalues should always be reported, either to support the use of a scree test or to show that it cannot be used.

Despite evident weaknesses in EFA, the subscales of earlier asthma specific HRQoL questionnaires reflect a common distinction of activity versus emotions, a distinction consistent with the theory that HRQoL judgements are affected by two causes [24]. One cause is the underlying pathology that creates disease specific symptoms and creates activity limitation, thereby creating the meaning dimension reflected in the My Life subscale. The other is the underlying personality of the patient which creates mood disturbance, thereby creating the meaning dimension reflected in the My Mind subscale. Similar activity versus emotion distinctions are found in subscales are based only on content [2, 4] and in those using statistical analysis [4–6]. In the case of severe asthma, however, there is an additional group of items and meaning dimension relating to the impact of non-asthma symptoms. These symptoms arise partly due to the polysymptomatic nature of severe asthma [25] and partly due to side effects caused by treatment such as oral corticosteroids. Treatment varies with severity but some patients experience more side effects than others. The three factor solution provides a disease specific set of subscales, subscales that are consistent with guidelines that questionnaires and their subscales should be fit for purpose [1], this being something that is not achieved with five or six factor solutions [4, 5].

The subscales differentiate between clinically relevant variables. The My Life subscale is more strongly related to lung function as measured by FEV_1_% predicted compared to the other two subscales, and the My Life subscale is also more strongly related to respiratory symptoms as measured by the ACQ or ACT scores when compared to the other two subscales. Poor lung function creates respiratory symptoms that then adversely affects daily activity, so these predictions are consistent with the hypothesis that respiratory symptoms are a major driver of activity limitation. It is possible that the My Life subscale is more sensitive to change in respiratory function in a clinical trial as activity subscales have been shown to be more sensitive to pharmaceutical interventions in two other asthma specific HRQoL scales [6, 26]. By contrast, the My Mind subscale is more strongly associated with participants’ response to a question on the severity of anxiety and depression compared to the other two subscales. This finding would indicate that the My Mind subscale may be most sensitive to change for interventions that affect mood. Finally, the content and statistical properties of the My Body subscale would indicate that this subscale may be most sensitive to changes in drug treatments that alter side effects and the experience of extra-pulmonary symptoms.

Quality of life and health are concepts used in clinical practice and research, but they are also words that are used by the general public in everyday speech. The SAQ asks people to evaluate the impact of asthma on ‘quality of life.’ The EQ5D asks people to rate their ‘health.’ Examination of Table 5 shows that both types of global estimate correlate most strongly with the My Life subscale compared to the other two subscales. When people are asked to make global estimates of ‘quality of life’ or ‘health’ they interpret these words and judge them by preferentially using the activity limitation construct as measured by the My Life subscale of the SAQ.

Although construct validity is an important part of subscale validation, the use of EFA for validating the overall score should be treated with caution. Items should not be selected on the basis of high factor loadings on a first factor as so doing can lead to overly restrictive set of items. Content validity through qualitative methods is an essential first step in establishing the items of a scale, as recommended by current guidelines [1]. Construct validation of subscales is carried out only after content validity is established. Although cross-loading items can be removed, such removal has the potential to weaken the breadth of the questionnaire.

A limitation of this study is that data were collected from only English speaking participants and not from participants responding to any of the validated translations of the SAQ. Those taking part were not randomly selected but selected by virtue of being under the care of a specialist severe asthma service. Estimated cumulative OCS dose is an estimate only. Additional longitudinal data collection is needed to establish the usefulness of subscales.

## Conclusions

There are two conclusions to be drawn from this study, one respiratory and the other methodological. The respiratory conclusion is that EFA provides evidence to interpret the items of the SAQ as clustering into three meaningful subscales, subscales that are linked to three different types of cause affecting severe asthma. The three SAQ subscales measure three different constructs or dimensions of meaning: impact on different life activities (measured by the My Life subscale), self-perceived mood disturbance (measured by the Mind subscale) and the impact of extra-pulmonary symptoms including side effects (measured by the My Body subscale). The three subscales provide a more nuanced picture of quality of life deficits that can be obtained from an overall score. The understanding provided by this more nuanced picture should help facilitate better communication between patient and healthcare workers and allow more detailed assessment regarding response to different treatments and management strategies, for example, whether an intervention reduces lifestyle limitations, improves mood, or reduces side effects—or does all three.

The methodological conclusion is that best practice guidelines for EFA that should be adopted in preference to default values in statistical packages, and that construct validation of HRQoL questionnaire subscales requires more than just running an EFA and reporting how items load on factors. The items of HRQoL questionnaires vary along many different dimensions of meaning. EFA is a way of showing the relationship between items and those meaning dimensions, and because of the complexity of meaning, the number of dimensions or number of factors selected in the EFA can vary depending on the degree of granularity of meaning required. The aim of EFA is to find a simple structure. That simple structure is ‘a’ plausible solution rather than necessarily ‘the’ solution, as more than one simple structure may be achievable. Guidelines for HRQoL require that the scale should be ‘fit for purpose’ [1] i.e., it should have a useful clinical role. The subscales of a HRQoL should therefore satisfy two constraints. First, the number and type of factors should be theoretically plausible and clinically relevant. Second the solution provided by EFA should be a simple structure where there is good separation between the factors. It may be that the wording of an item represents meaning in two or more important meaning dimensions (e.g., item 14 in the SAQ), and this will be represented by cross-loading of that item. Cross-loading items are undesirable because they are poor discriminators between subscales. Valid subscales are made up from items that, with few exceptions, do not cross-load. However, an HRQoL scale will not have valid subscales if the only way to avoid cross-loading and achieve simple structure is to have a large number of factors with doubtful clinical use made up from few (less than three) items in some of the factors. Not all HRQoL scales will have construct valid subscales. This paper provides a template for future use of EFA for establishing validity of subscales of HRQoL questionnaires.

## Data Availability

The data set can be obtained from the corresponding author or from Joseph Lanario (joseph.lanario@plymouth.ac.uk) either in Excel or SPSS format.
